# Automated gathering of real-world data from online patient forums can complement pharmacovigilance for rare cancers

**DOI:** 10.1038/s41598-022-13894-8

**Published:** 2022-06-20

**Authors:** Anne Dirkson, Suzan Verberne, Wessel Kraaij, Gerard van Oortmerssen, Hans Gelderblom

**Affiliations:** 1 LIACS, Leiden University, Niels Bohrweg 1, 2333 CA Leiden, The Netherlands; 2 Patient Platform Sarcomas, Utrecht, The Netherlands; 3 Department of Medical Oncology, Leiden University Medical Centre, Albinusdreef 2, 2333 ZA Leiden, The Netherlands

**Keywords:** Sarcoma, Software, Quality of life, Drug regulation

## Abstract

Current methods of pharmacovigilance result in severe under-reporting of adverse drug events (ADEs). Patient forums have the potential to complement current pharmacovigilance practices by providing real-time uncensored and unsolicited information. We are the first to explore the value of patient forums for rare cancers. To this end, we conduct a case study on a patient forum for Gastrointestinal Stromal Tumor patients. We have developed machine learning algorithms to automatically extract and aggregate side effects from messages on open online discussion forums. We show that patient forum data can provide suggestions for which ADEs impact quality of life the most: For many side effects the relative reporting rate differs decidedly from that of the registration trials, including for example cognitive impairment and alopecia as side effects of avapritinib. We also show that our methods can provide real-world data for long-term ADEs, such as osteoporosis and tremors for imatinib, and novel ADEs not found in registration trials, such as dry eyes and muscle cramping for imatinib. We thus posit that automated pharmacovigilance from patient forums can provide real-world data for ADEs and should be employed as input for medical hypotheses for rare cancers.

## Introduction

Adverse Drug Events (ADEs), harmful reactions that result from the intake of medication, pose a major health concern^1^ and can have a great impact on the quality of life of a patient^2^. Clinical trials are unable to fully assess the ADEs of a drug due to their limited duration and relatively small sample size, which precludes the discovery of long-term ADEs and rarer ADEs. Furthermore, clinical trials focus on patients in relatively good condition. They mostly exclude elderly, patients with comorbidities, pregnant women, and children^3,4^, and thereby are unable to assess the ADEs that may occur within these patient groups.

Despite post-market surveillance systems, ADEs remain severely under-reported with on average over 90% of ADEs remaining undiscovered^5^. Especially non-serious ADEs are under-reported despite the strong influence they might have on patient adherence and quality of life (QoL)^6^.There is an increased recognition that information sources that are more representative of the everyday ‘real world’ are necessary to supplement clinical trials^7,8^. In recent years, both the FDA and EMA have started to investigate how they can make use of such real world evidence to strengthen their post-market surveillance of drugs (i.e. pharmacovigilance)^9^. One promising resource for the semi-automatic discovery of real-world evidence is social media data^10–12^.

The main advantage of using social media for pharmacovigilance is that it is uncensored and spontaneous. Previous studies have shown that the attitudes of medical professionals cause bias in ADE reporting. Surveys show that medical professionals may not report an ADE for various reasons including lack of time, uncertainty about whether the drug causes the ADE or because the ADE is either trivial or well-known^13,14^. Social media data has several other distinct advantages compared to other potential information sources. First, the sheer volume of information is not easily obtainable by other means^15^. Second, it has been found that users more often share information with peers than with physicians or at clinical trials^16^. A third advantage is that social media is able to provide near-instantaneous information which allows for real-time monitoring and early signal detection^17^. Yet, some concerns of representativeness of users and data quality have also been put forward^18,19^ which we will address in the discussion.

Patient forums, online communities where patients gather to exchange information and experiences, are a type of social media that could be especially valuable as a resource for ADE detection. It has been estimated that 8% of posts in specific online forums for patients are reports of adverse drug events^20^. Nonetheless, most research at present has focused on generic social media^15,21^. In this article, we present the first empirical case study investigating the value of automated pharmacovigilance from patient forums for a rare cancer. In collaboration with patient organizations, we have collected and extracted ADEs from a large forum of patients with Gastro-Intestinal Stromal Tumors (GIST). Although it is the most common of the sarcomas, it is a rare disease with an incidence of 10–15 per million per year^22^.

## Materials and methods

### Data collection

In agreement with the GIST International Support Organization, we collected data from their at the time public Facebook group using the Facebook API. The data ranges from 24 Oct 2009 until 1 Nov 2020 and includes 121,561 English messages in 14,631 conversational threads. The 1,493 non-English messages (1.2%) on the forum were removed. On 1 Nov 2020, the forum had 5,555 members and 1567 users were active on that day. Our study design and data management plan were approved by the Leiden University privacy officer. We did not collect usernames to protect user privacy in line with data minimization practices. The collected messages were stored securely, and access was restricted to the involved researchers and annotators. For the labelling of data, we did not use commercial tools but set up private servers that were only accessible to the annotators. In accordance with the GDPR (Article 9.2), we did not obtain consent from each user as the GDPR allows for the use of data from publicly accessible forums with justified cause without individual consent. The necessity to take informed consent was formally waived by the Leiden University privacy officer. Nonetheless, we are unable to share the data according to the GDPR, because access to the forum has become restricted to members since our data collection (i.e., it is no longer publicly accessible).

### Machine learning pipeline

**Figure 1:**
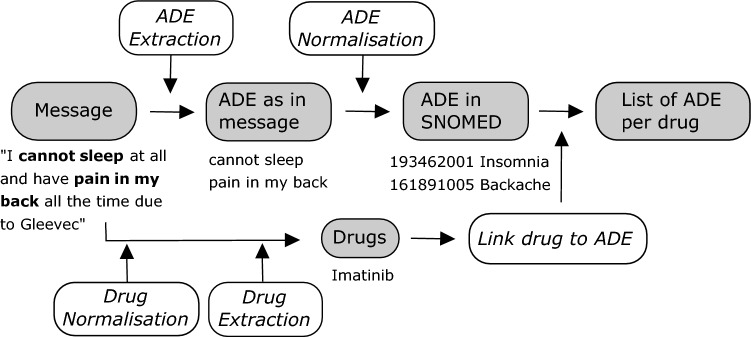
An overview of the software pipeline we developed for automatically determining which adverse drug effects (ADE) are mentioned on a patient forum. All italicized parts indicate modules we developed. An example message is provided to clarify each step. ADE: adverse drug events.

We developed a software pipeline to automatically extract the ADEs from the messages on the patient forum using state-of-the-art methods. As shown in Fig. 1, we first extract (i.e., ADE Extraction) the words that contain an ADE (e.g., ‘cannot sleep’) from each message using a specialized information extraction model. This model is trained on forum messages that are manually labelled for ADEs by human annotators. For such tasks where words that contain a certain concept (like an ADE) are extracted (also called Named Entity recognition tasks), predictions are done for each individual word in the sentence. So, the data for training this model is also labelled per word. Specifically, words are labelled for if they are at the Beginning of an entity (B), Inside an entity (I) or Outside an entity (O)^23^. This is the most common format for sequence labelling tasks, or tasks in which predictions are made per word. Forum messages can contain multiple ADE, which may also span across sentences.

Since posts that contain ADE are a small subset of the data, we wanted to select posts that had a high likelihood to contain an ADE to reduce the time the annotators needed to spend on labelling the data before we had sufficient manually labelled examples to train our model. To create our data selection for manual labelling, we selected all discussions that contained at least one drug name (i.e. one exact match with a drug in RxNORM^24^). Prior to data selection, drug names were normalized to their generic variants (e.g., Gleevec to imatinib) and spelling correction was applied to correct misspelt drug names (see Appendix A.1 for more details on preprocessing). From the discussion threads with at least one drug name, we selected the discussions with the highest percentage of posts in which authors shared experiences (such as that you experienced an ADE). In order to estimate which percentage of the posts in a thread included patient experiences, we used a previously developed model^25^. In short, the model was a linear SVC classifier based on trigrams (i.e., sequences of three letters) that could identify experiences with an overall performance (F_1_ score) of 0.815.

In total, 4195 messages (527 discussions) from the GIST forum which were labelled by three GIST patients and the first author using an annotation guideline(Available at https://github.com/AnneDirkson/ConversationAwareFiltering/tree/master/guideline). Subsets of the data (30 threads, between 179 to 211 posts total) were annotated by two annotators to be able to measure to what extent they would label the data the same. Each annotator would label two such overlapping sets. We choose to not have all annotators label the same overlapping data to decrease their workload. For our data, the average agreement between two human annotators was substantial (mean Cohen’s κ = 0.71). A small sample of the annotated data is available as a Supplementary File as an example.

We use 80% of our annotated data and an additional 1,250 messages from a publicly available data set^26^ to train our model. Another 10% of our annotated data is used to determine how we can best train our model (i.e., the development data). See Section A.2 for the technical details on how we trained our extraction model and Section A.1 for details on how the data was preprocessed(i.e. transformed from raw data to input for a machine learning model) before ADE extraction. The remaining 10% of the annotated data is used to evaluate how well our model works on data it has not seen before (i.e., the test data).

We find that on this test data our model has a sensitivity (also called recall) of 0.739: it can retrieve 52.3% of entities fully and 16.6% partially. If it retrieves an entity partially, it has managed to label some of the words of the entity correctly but not all. The specificity of the model is 0.998, meaning that it can correctly identify 99.8% of the true negatives. Its precision of the model is 0.695, meaning that 69.5% of all retrieved entities are true positives. Our model thereby outperforms state-of-the-art models on this task^27^. Yet, its overall performance (F_1_ = 0.72) is still slightly lower than that of humans (average pair-wise F_1_ = 0.80). Moreover, we find that our model is able to find new adverse drug events for which there were no manually labelled examples (see Section A.2 for more detail).

We use a specialized machine learning model to link the extracted phrases containing ADE (e.g., ‘cannot sleep’) to concepts in SNOMED-CT (e.g., Insomnia) (i.e., ADE Normalization in Fig. 1). This allows us to aggregate instances where the same ADE is expressed in different ways. In general terms, this model compares the extracted ADE to all synonyms of concepts in a selected subset of SNOMED to find the best match by ranking how similar each synonym is to the extracted ADE. We train this model using three external data sets^26,28,29^. On average, this model can correctly label 64.5% of the ADEs. For an additional 14.6% of the cases, the correct label was included in the top 5. See Section A.3 for more details on the training and evaluation of the normalization model.

We also extract the medication mentioned in the forum message. We first change all medication names to their generic forms (e.g., Gleevec to Imatinib) during Drug Normalization. For this step, we use the RxNORM database^24^. We then extract all the generic drug names (e.g., Imatinib) during Drug Extraction using a list of generic drug names from the RxNORM. Finally, we determine which drug the ADE mentioned in the message is most likely to belong to, based on the message and the conversational thread (i.e., Link drug to ADE in Fig. 1). We designed a simple set of rules (see Section A.4) that select the correct drug 93% of the time if we restrict the possible choices to a list of possible GIST medications (i.e. Imatinib, Sunitinib, Regorafenib, Avapritinib, Ripretinib, Nilotinib, Pazopanib, Ponatinib, Sorafenib)to prevent drugs that resolve the ADE (e.g., ‘ondansetron’ for nausea) from being not chosen. An ADE is linked to no drug (‘Unknown’) if no drug is mentioned in the message nor in the conversational thread prior to the message.

For the purpose of follow-up research, we describe all technical details of our pipeline in the Appendix A, and we have made our code open-source (https://github.com/AnneDirkson/CHyMer). Our pipeline for ADE extraction from patient forums is the first that is both publicly available and targeted at English data. Van Stekelenborg et al*.*^30^ employed proprietary software and the work by Audeh et al*.*^10^ is on French data. Although we are unable to share the original forum messages, we provide an output file of all extracted ADEs (including which drug they are linked to) for each discussion thread and post as a Supplementary File.

### Data analysis

We investigate the ADEs reported online for all medication that is standard treatment for GIST patients: the first-line treatment imatinib, the second-line treatment sunitinib, the third-line treatment regorafenib, and two recently approved drugs, namely ripretinib, now fourth line treatment, and avapritinib, which was specifically approved for PDGFRA exon 18 mutations. Both were approved in 2020^31,32^. All analyses were conducted in Python.

We first identify the 20 most prevalent ADEs for each drug. It is important to note that if an ADE was mentioned twice in one message, it was counted only once. Due to privacy considerations, we do not have access to data on who posted which message and consequently, we are unable to remove cases where the same person posts about an ADE multiple times in different messages. We aggregate ADEs into categories based on the SNOMED-CT hierarchy and the medical expertise of Prof. Dr. Gelderblom.

We also inspect long-term ADEs for GIST medication that has been on the market for more than five years (i.e., imatinib, sunitinib and regorafenib). We define long-term ADEs as ADEs that have their first mention on the forum after more than five years of ADE reports concerning that particular drug on the forum. We thereby assume that short-term ADEs will be mentioned at least once in the first five years of ADE reports for a particular drug. Note that we use this proxy because we do not have information on how long patients posting on the forum have been taking a drug as we do not know who posted a message. A limitation of our approach is that rare (but not necessarily long-term) ADEs may not be filtered out. However, by considering how frequently long-term ADEs are reported, we can partially mitigate this issue. We do not aggregate ADEs into larger categories for this analysis because we found that this favored categories containing very many infrequently occurring ADEs over more relevant ADE. For the 20 most prevalent long-term ADEs, we manually checked whether there were erroneous categories of ADE that were the result of errors during the extraction step (e.g., ‘elevated mood’ was assigned to any case in which only ‘elevated’ was extracted instead of the full ADE).

Finally, we investigate which ADEs mentioned on the forum were not reported in the registration trials. We compare our findings to the registration trials for GIST patients instead of the general Summary of Product Characteristics (SmPC) of the drug because the SmPC is not specific to our patient population whereas the registration trials are. For imatinib, we included one phase II trial^33^, two phase III trials^34,35^ for Gastrointestinal Stromal Tumor patients based on the approval summary^36^ and the work by Reichardt^37^. We also include the ADEs mentioned for GIST in the FDA report for imatinib^38^. For sunitinib, we include one phase III trial for GIST^39^ and ADEs mentioned for GIST in the FDA report^40^. For regorafenib, we include one phase III trial for GIST^41^ and the ADEs for GIST in the FDA report^42^. We provide supplementary files describing which specific ADEs (with their manually assigned SNOMED CT identifier) were included for each medication.

For this analysis, we set a threshold of 5 as a minimum frequency (i.e., the ADE needed to be mentioned on the forum at least 5 times). We first automatically filtered out any ADEs that were mentioned in the registration trial using their SNOMED-CT identifier. We also filtered out all SNOMED concepts that occurred below these concepts in the SNOMED hierarchy (e.g., leg edema falls under edema and should also be filtered out). Prof. Dr. Gelderblom then manually verified the most prevalent novel ADEs for each drug by comparing them to the ADEs mentioned in registration trial. We also manually removed any ADE categories from the top 20 that were fully the result of extraction errors.

## Results

Table 1 reports the number of ADEs found for each medication type on the GIST patient forum. The amount of ADEs reported increases with the number of patients that have been prescribed a certain medication. Manual analysis revealed that most of the ‘Unknown’ cases are in fact not ADEs but symptoms of GIST or side effects of surgery.

**Table 1:** The number of ADEs found for each medication type on the GIST patient forum.

Treatment type	Drug	# of ADE found	# of ADE types
First-line	Imatinib	13,376	685
Second-line	Sunitinib	2,335	324
Third-line	Regorafenib	319	226
Fourth-line	Ripretinib	319	90
PDGFRA exon 18 mutations	Avapritinib	297	112
Off-label	Nilotinib	59	40
Off-label	Pazopanib	51	27
Off-label	Sorafenib	47	32
Off-label	Ponatinib	17	13
	Unknown	2948	497
	Total	21,051	1,086

**Figure 2:**
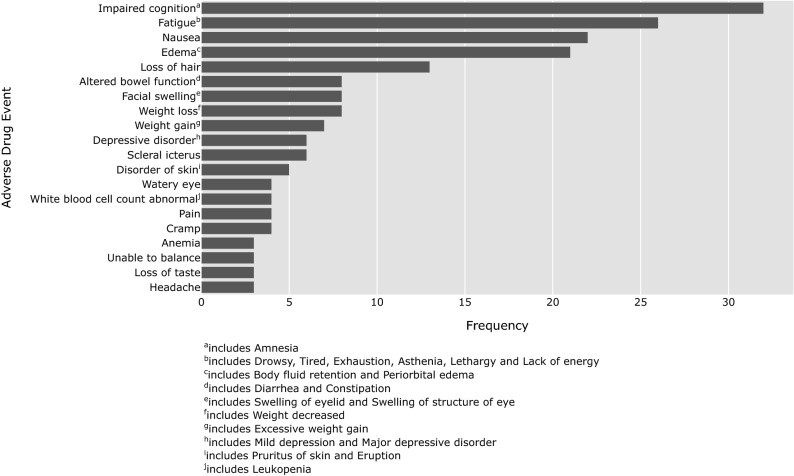
The 20 most prevalent adverse drug events reported for avapritinib (formerly BLU-285) on the GIST patient forum.

**Figure 3:**
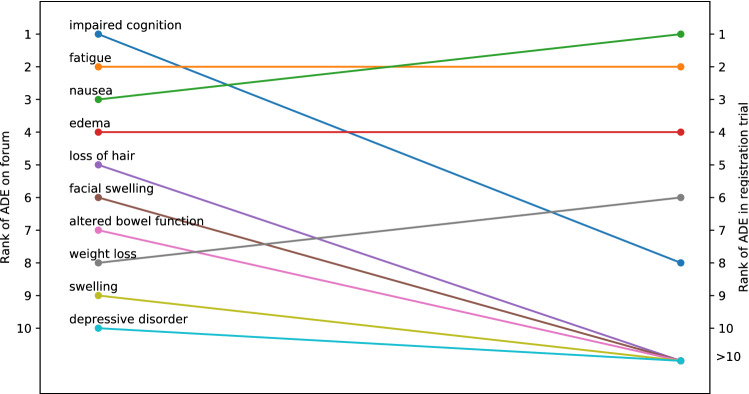
The change in rank in terms of prevalence of reporting of the top 10 adverse drug events found for avapritinib on the forum (left) compared to the registration trial (right). ADE: adverse drug events.

For each medication, we can analyze how often ADEs are reported. For example, Fig. 2 shows the most often reported ADEs for avapritinib. Impaired cognition is the most reported ADE followed by fatigue, nausea, edema, and loss of hair. These ADEs were all reported in the registration trial albeit in the different order as can be seen in Fig. 3 (e.g., cognitive impairment was the 8th most prevalent ADE in the registration trial). Incidence rates of ADEs from the clinical trials cannot be compared to the relative reporting ratesof ADEs on the forum directly, as nonclinical social media data does not allow us to infer who does *not* have an ADE. Users that do not report an ADE might still experience it. Thus, reporting rates of ADEs from forum data are only interpretable in a relative sense (i.e., nausea is reported more than fatigue). Nonetheless, relative differences between ADE reporting on a forum and incidence from the registration trial can provide insight into which ADEs are perceived by patients as having the most negative impact on their quality of life; ADEs that are reported relatively more often than expected based on incidence are more salient to patients. Aside from cognitive impairment, we find that, for example, loss of hair (i.e., alopecia) is reported more often than one would expect based on the prevalence in the clinical trial. It was in fact the 23rd or least prevalent ADE at 13% of all patients.

**Figure 4:**
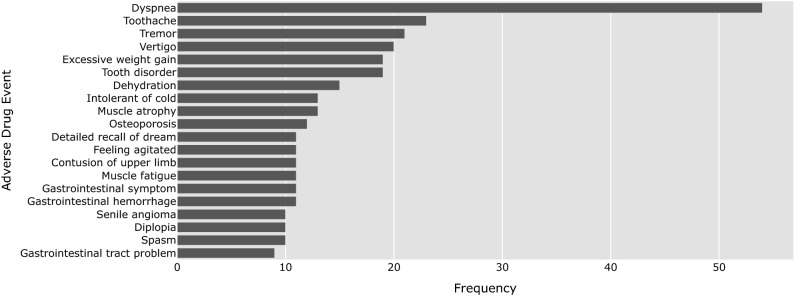
The 20 most prevalent long-term adverse drug events reported for imatinib on the forum.

We also analyze ADEs that occur after long-term use of a drug. Figure 4 shows the most prevalent long-term ADEs reported for Imatinib on the GIST patient forum. The most reported are dyspnea, toothache, tremor, vertigo and excessive weight gain. It appears that patients suffer from problems with their teeth (i.e., toothache and tooth disorder), muscles (i.e., tremor, muscle atrophy and muscle fatigue), and skeletal system (i.e., osteoporosis). We acknowledge that these ADEs might be related to other factors such as age, and no definitive causality can be deduced from patient reports. Nonetheless, analysis of long-term ADEs on patient forums can provide valuable indications of directions for further investigation.

**Figure 5:**
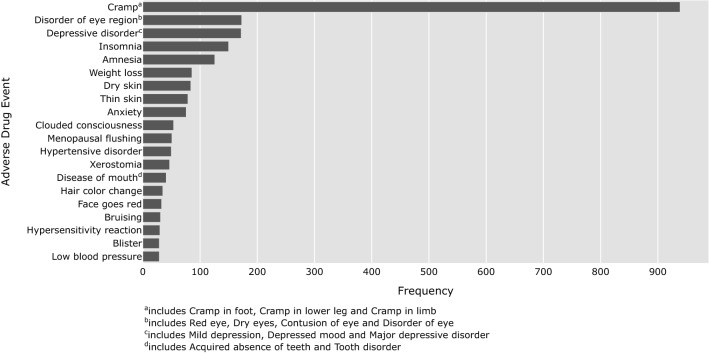
The 20 most prevalent adverse drug events for imatinib that were not found in the registration trials.

Finally, we compare the ADEs found in registration trials to those reported on the GIST patient forum to uncover novel ADEs for GIST patients. In contrast to generic social media, disease-specific forums have the unique benefit of providing ADEs for a specific patient population, e.g., GIST patients. In turn, this enables the comparison to known ADEs for that specific patient population through comparison with the relevant clinical trials. For imatinib, we initially found 214 novel ADEs that were reported at least 5 times. Figure 5 shows the 20 most prevalent ADEs reported for imatinib that were not reported in the registration trials (the list was curated by an oncologist specialized in sarcomas). Muscle cramp, problems with the eyes, depression, insomnia and amnesia are reported most often. Patients also report novel skin problems (i.e., dry skin, thin skin, bruising and blisters), mouth problems (i.e., xerostomia and tooth problems) and problems with too high or low blood pressure.

Although these ADEs had not been reported during the registration trials for use of imatinib for GIST, many are included in the general Summary of Product Characteristics (or SmPC) of imatinib^43^, which means that they have either been found for another disorder (e.g., imatinib is also used by patients with chronic myelogenous leukemia (CML)) or that they were found in the post-marketing phase. Overlap between the SmPC and the 20 most prevalent ADEs that were not reported in the registration trials includes muscle cramps, eye disorders, depression, insomnia, amnesia, weight loss, dry skin, anxiety, high and low blood pressure, xerostomia (dry mouth), bruising and blisters. For ADEs found for other disorders, forum data can provide an indication that these ADEs also occur amongst GIST patients. A high degree of overlap with other patient populations taking imatinib is not surprising, as many ADEs may not be disease-specific. Adverse drug events may also have been added to the SmPC as a result of post-marketing reports by GIST patients. Overlap with these ADEs is promising, as it underscores that forum data may pose an alternative for obtaining such information after release of a drug onto the market.

Forum data can also indicate ADEs that are novel for all imatinib users. Thin skin, clouded consciousness, menopausal flushing, change in hair color, and tooth problems are examples of adverse drug events found on the forum that were not reported in either registration trials for GIST or in the general SmPC.

For the purpose of more detailed investigations, we provide an interactive demo for clinical researchers to access all analyses at: https://dashboard-gist-adr.herokuapp.com/.

## Discussion

In this article, we showcase the potential of patient forums as a complementary source of knowledge for pharmacovigilance for rare cancers with a case study. Although ADEs mentioned on a patient forum provide valuable information, causality assessment is necessary before this information can be used as real-world evidence. Similar to spontaneous reporting through official channels, the causality of an adverse drug event needs to be determined before it can be coined an adverse drug response. Whereas an adverse drug event is “any untoward (i.e., unexpected and negative) medical occurrence that may appear during treatment with a pharmaceutical product but which does not necessarily have a causal relationship with the treatment”, an adverse drug response infers a causality relation between drug and effect^44,45^.

Our work differs from previous studies^10,30^ in a number of important aspects. First, in contrast to previous work, we assess ADEs in the context of a specific disease. This enables us to compare our results to registration trials specific to that patient population. We believe that this approach is far more promising than previous approaches which assess ADEs irrespective of which patients are taking the drug, as our approach allows for an investigation of the value of pharmacovigilance from patient forums for specific diseases, including rare and orphan diseases.

We assessed which ADEs are novel in comparison to those found in the registration trial prior to market release. Thus, we did not take into account which ADEs are discovered by official post-marketing systems, such as by the FDA or EMA, for GIST patients. These systems do not share with researchers which patients reported which ADE and thus all ADEs for a drug are aggregated irrespective of disorder. Comparisons to a specific patient population are thus not possible at this time, although such comparisons would be valuable. There are promising initiatives such as OHDSI (https://ohdsi.org/) that are attempting to make such detailed analysis possible in the future.

The focus on rare disorders is the second major difference with previous work. Semi-automatic discovery of ADEs from patient forums is particularly promising for patients with rare diseases, because clinical research into these disorders is scarce. This lack of research is due to a combination of low funding, low interest from pharmaceutical companies, and dispersed patient communities^46–48^. In fact, according to Aymé et al.^46^ online forums could enable the coordinated, trans-geographic effort that is necessary to attain progress for rare diseases.

Moreover, we are the first study to investigate automatic extraction of long-term side effects from online forums. Some GIST patients take imatinib for longer than 5 or 10 years due to its efficacy^49,50^. Although post-market clinical studies have evaluated the long-term efficacy of imatinib^49,50^, only one study^49^ recorded adverse events and only if they were the reason patients reduced their dosage. The ADEs reported were edema, fatigue, rash and diarrhea. These ADEs were also reported in the original registration trial and are consequently not specific to long-term usage.

Despite the promise of patient forums as a resource for real-world data, two sources of concern have also been expressed in the literature. A first concern is that the patients that post on the patient forum are not representative for the general patient population^18,19^. Some patients may lack the skills, access or desire to post on social media^51^. Generally speaking, young people, women and those of higher socioeconomic class are more highly represented on social media^19^. To address this concern, our future work will include a survey amongst GIST patients to investigate the representativity bias on patient forums. Furthermore, this concern is not in fact unique to social media as a potential resource for pharmacovigilance; Clinical trials, surveys and spontaneous reports are also subject to representativity bias. A second concern that has been posited is that the quality of the ADE reports from social media may be inferior. However, studies have shown that reports from patients can be similar in quality compared to those of healthcare professionals^52^. This is also the case for reports on patient forums^53^.

Nonetheless, our method does have some limitations due to three sources of noise. Automatic extraction using machine learning methods enables the processing of large volumes of forum messages but also introduces errors into the data as methods do not attain perfect performance e.g., reports may be missed, false positives may be included, or ADEs may be linked to the wrong concept (see Appendix A.3 for a more detailed evaluation of errors). A second possible source of noise is negated ADEs, i.e., when a user indicates they do not have a certain ADE. We do not separately identify whether an ADE is negated, because our model is only trained to recognize cases where the ADE is not negated using labeled data in which only non-negated ADE are annotated. However, our model may erroneously extract negated ADE, as they are textually similar to true positives. Furthermore, duplicate records in the data may also introduce noise. Patients may post multiple times about the same ADE and since we do not have access to (anonymized) usernames of posters, we cannot remove these duplicates. Consequently, the real-world data provided by patient forums is noisier overall than the data obtained from spontaneous reports or clinical trials. Automatically extracted ADEs from patient forums should be interpreted in this light; Individual reports may be less reliable but on an aggregate level these reports can provide valuable indications of ADEs and issues that patients are facing. Further clinical research or surveys could be used to validate these hypotheses.

## Conclusion

In this article, we have shown with a case study of an online forum for GIST patients that patient forums can provide real-world data for both long-term ADEs, such as osteoporosis and tremors for imatinib, as well as for ADEs that were not found in the original registration trials, such as dry eyes and muscle cramping for imatinib. Patient forums are also able to reveal a patient-centric perspective of ADEs by showing which ADEs affect quality of life the most. We find that the relative reporting rate of an ADE often differs decidedly from that of the registration trials. For example, alopecia and cognitive impairment were both reported far more often for avapritinib than would have been expected based on the prevalence in the registration trial. Thus, despite its limitations and noisy nature, automated extraction of ADEs from patient forums can help combat current under-reporting of ADEs by providing much needed real-world data that can function as input for new medical hypotheses and research.

## Supplementary Information

Supplementary Information 1.

Supplementary Information 2.

Supplementary Information 3.

Supplementary Information 4.

Supplementary Information 5.

Supplementary Information 6.

## Data Availability

The data are not publicly available due to the protection of privacy of the patients under the GDPR, because access to the forum has become restricted to members since our data collection (i.e., it is no longer publicly accessible). Our study design and data management plan were approved by the Leiden University privacy officer. The necessity to take informed consent was formally waived by the Leiden University privacy officer under GDPR article 9.2. We make two data sets available as supplementary material. The first (Extracted_ADE_forum.tsv) is a comprehensive table containing if adverse drug events were found for each discussion thread and post in our data, and if so, which one (Concept ID from the ontology and Default concept name). It also contains the information which drug the extracted ADEs were linked to. The original post as well as the extracted phrase has been omitted in line with the GDPR. In addition, we supply an example of our data format: one annotated discussion thread to show how the data was annotated (Annotated_discussion_thread_example.tsv).

## Code availability

The code is available at https://github.com/AnneDirkson/CHyMer.

## References

[1] World Health Organisation, The Safety of Medicines in Public Health Programmes: Pharmacovigilance an essential tool, 2006.

[2] Rolfes L, van Hunsel F, van der Linden L, Taxis K, van Puijenbroek E (2017). The quality of clinical information in adverse drug reaction reports by patients and healthcare professionals: A retrospective comparative analysis. Drug Saf..

[3] Shenoy P, Harugeri A (2015). Elderly patients’ participation in clinical trials. Perspect. Clin. Res..

[4] Stricker BH, Psaty BM (2004). Detection, verification, and quantification of adverse drug reactions. BMJ.

[5] Hazell L, Shakir SAW (2006). Under-reporting of adverse drug reactions a systematic review. Drug Saf..

[6] Rolfes L, van Hunsel F, Taxis K, van Puijenbroek E (2016). The impact of experiencing adverse drug reactions on the patient’s quality of life: A retrospective cross-sectional study in the Netherlands. Drug Saf..

[7] Plueschke K, McGettigan P, Pacurariu A, Kurz X, Cave A (2018). EU-funded initiatives for real world evidence: Descriptive analysis of their characteristics and relevance for regulatory decision-making. BMJ Open.

[8] Klonoff DC, Gutierrez A, Fleming A, Kerr D (2019). Real-world evidence should be used in regulatory decisions about new pharmaceutical and medical device products for diabetes. J. Diabetes Sci. Technol..

[9] Radawski, C. A. *et al.*, The utility of real‐world evidence for benefit‐risk assessment, communication, and evaluation of pharmaceuticals: Case studies, *Pharmacoepidemiol. Drug Saf.*, p. pds.5167 (2020). 10.1002/pds.5167 33146901

[10] Audeh B, Bellet F, Beyens MN, Lillo-Le Louët A, Bousquet C (2020). Use of social media for pharmacovigilance activities: Key findings and recommendations from the project. Drug Saf..

[11] S. Golder, K. Smith, K. O’Connor, R. Gross, S. Hennessy, and G. Gonzalez-Hernandez, A comparative view of reported adverse effects of statins in social media, regulatory data, drug information databases and systematic reviews, *Drug Saf.*, 1–13 (2020). 10.1007/s40264-020-00998-1 PMC7847442 33001380

[12] S. Khosla *et al.*, Real world evidence (RWE)—a disruptive innovation or the quiet evolution of medical evidence generation?, *F1000Research*, 7, 111, 2018. 10.12688/f1000research.13585.1 PMC6039945 30026923

[13] Harpaz R, DuMouchel W, Shah NH, Madigan D, Ryan P, Friedman C (2012). Novel data-mining methodologies for adverse drug event discovery and analysis. Clin. Pharmacol. Ther..

[14] Eland IA, Belton KJ, Van Grootheest AC, Meiners AP, Rawlins MD, Stricker BHC (1999). Attitudinal survey of voluntary reporting of adverse drug reactions. Br. J. Clin. Pharmacol..

[15] Sarker A (2015). Utilizing social media data for pharmacovigilance: A review. J. Biomed. Inform..

[16] Davison KP, Pennebaker JW, Dickerson SS (2000). Who talks? The social psychology of illness support groups. Am. Psychol..

[17] Sloane R, Osanlou O, Lewis D, Bollegala D, Maskell S, Pirmohamed M (2015). Social media and pharmacovigilance: A review of the opportunities and challenges. Br. J. Clin. Pharmacol..

[18] Bousquet C (2017). The adverse drug reactions from patient reports in social media project: Five major challenges to overcome to operationalize analysis and efficiently support pharmacovigilance process. JMIR Res. Protoc..

[19] Cesare, N., Grant, C. & Nsoesie, E. O. Understanding demographic bias and representation in social media health data, in *WebSci 2019—Companion of the 11th ACM Conference on Web Science*, 2019, pp. 7–9.

[20] Golder S, Norman G, Loke YK (2015). Systematic review on the prevalence, frequency and comparative value of adverse events data in social media. Br. J. Clin. Pharmacol..

[21] Lardon J (2015). Adverse drug reaction identification and extraction in social media: A scoping review. J. Med. Internet Res..

[22] Søreide K, Sandvik OM, Søreide JA, Giljaca V, Jureckova A, Bulusu VR (2016). Global epidemiology of gastrointestinal stromal tumours (GIST): A systematic review of population-based cohort studies. Cancer Epidemiol..

[23] Ramshaw LA, Marcus MP, Armstrong S, Church K, Isabelle P, Manzi S, Tzoukermann E, Yarowsky D (1999). Text chunking using transformation-based learning. Natural language processing using very large corpora.

[24] U.S. National Library of Medicine, “RxNorm.” [Online]. Available: https://www.nlm.nih.gov/research/umls/rxnorm/.

[25] A. Dirkson, S. Verberne, and W. Kraaij, “Narrative Detection in Online Patient Communities,” in *Proceedings of the Text2StoryIR’19 Workshop*, 2019.

[26] Karimi S, Metke-Jimenez A, Kemp M, Wang C (2015). Cadec: A corpus of adverse drug event annotations. J. Biomed. Inform..

[27] Weissenbacher, D. *et al.*, Overview of the Fourth Social Media Mining for Health (#SMM4H) Shared Task at ACL 2019, in *Proceedings ofthe 4th Social Media Mining for Health Applications (#SMM4H) Workshop & Shared Task*, 2019, pp. 21–30.

[28] Basaldella, M., Liu, F., Shareghi, E., & Collier, N. COMETA: A Corpus for Medical Entity Linking in the Social Media, in *Proceedings of the 2020 Conference on Empirical Methods in Natural Language Processing*, 2020, pp. 3122–3137.

[29] Zolnoori, M. *et al.*, “The PsyTAR dataset: From patients generated narratives to a corpus of adverse drug events and effectiveness of psychiatric medications.,” *Data Br.*, vol. 24, Jun. 2019. 10.1016/j.dib.2019.103838 PMC6495095 31065579

[30] van Stekelenborg J (2019). Recommendations for the Use of Social Media in Pharmacovigilance: Lessons from IMI WEB-RADR. Drug Saf..

[31] FDA, “FDA approves ripretinib for advanced gastrointestinal stromal tumor,” 2020. [Online]. Available: https://www.fda.gov/drugs/drug-approvals-and-databases/fda-approves-ripretinib-advanced-gastrointestinal-stromal-tumor.

[32] European Medicine Agency, “Ayvakyt,” 2020. [Online]. Available: https://www.ema.europa.eu/en/medicines/human/EPAR/ayvakyt.

[33] Demetri GD (2002). Efficacy and safety of imatinib mesylate in advanced gastrointestinal stromal tumors. N. Engl. J. Med..

[34] Verweij J (2004). Progression-free survival in gastrointestinal stromal tumours with high-dose imatinib: Randomised trial. Lancet.

[35] Blanke CD (2008). Phase III randomized, intergroup trial assessing imatinib mesylate at two dose levels in patients with unresectable or metastatic gastrointestinal stromal tumors expressing the kit receptor tyrosine kinase: S0033. J. Clin. Oncol..

[36] Dagher R (2002). Approval summary: imatinib mesylate in the treatment of metastatic and/or unresectable malignant gastrointestinal stromal tumors. Clin. Cancer Res..

[37] Reichardt P (2018). The story of imatinib in GIST-a journey through the development of a targeted therapy. Oncol. Res. Treat.

[38] U.S. Food and Drug Administration (FDA), “GLEEVEC (imatinib mesylate) tablets Label.” [Online]. Available: https://www.accessdata.fda.gov/drugsatfda_docs/label/2008/021588s024lbl.pdf.

[39] Demetri GD (2006). Efficacy and safety of sunitinib in patients with advanced gastrointestinal stromal tumour after failure of imatinib: A randomised controlled trial. Lancet.

[40] U.S. Food and Drug Administration (FDA), “SUTENT (sunitinib malate) capsules label.” [Online]. Available: https://www.accessdata.fda.gov/drugsatfda_docs/label/2011/021938s13s17s18lbl.pdf.

[41] Demetri GD (2013). Efficacy and safety of regorafenib for advanced gastrointestinal stromal tumours after failure of imatinib and sunitinib (GRID): An international, multicentre, randomised, placebo-controlled, phase 3 trial. Lancet.

[42] U.S. Food and Drug Administration (FDA), “STIVARGA (regorafenib) tablets label.” [Online]. Available: https://www.accessdata.fda.gov/drugsatfda_docs/label/2017/203085s007lbl.pdf.

[43] E. M. Agency, Summary of Product Characteristics Imatinib. [Online]. Available: https://www.ema.europa.eu/en/documents/product-information/glivec-epar-product-information_en.pdf.

[44] World Health Organization, The Importance of Pharmacovigilance: Safety Monitoring of medicinal products, Geneva, 2002.

[45] European Medicine Agency, Guideline on good pharmacovigilance practices (GVP) - Annex I - Definitions (Rev 4), 2017.

[46] Aymé S, Kole A, Groft S (2008). Empowerment of patients: Lessons from the rare diseases community. Lancet.

[47] Heemstra HE, van Weely S, Büller HA, Leufkens HGM, de Vrueh RLA (2009). Translation of rare disease research into orphan drug development: Disease matters. Drug Discov. Today.

[48] U.S Congress Office of Technology Assessment, *Pharmaceutical R\&D: Costs, Risks, and Rewards*. Washington, DC: U.S. Government Printing Office, 1993.

[49] Ogata K (2018). Long-term imatinib treatment for patients with unresectable or recurrent gastrointestinal stromal tumors. Digestion.

[50] Casali PG (2017). Ten-year progression-free and overall survival in patients with unresectable or metastatic GI stromal tumors: Long-term analysis of the european organisation for research and treatment of cancer, Italian sarcoma group, and Australasian gastrointestinal tr. J. Clin. Oncol..

[51] Price J (2016). What Can Big Data Offer the Pharmacovigilance of Orphan Drugs?. Clin. Ther..

[52] Blenkinsopp A, Wilkie P, Wang M, Routledge PA (2007). Patient reporting of suspected adverse drug reactions: A review of published literature and international experience. Br. J. Clin. Pharmacol..

[53] van Uden-Kraan CF (2008). Coping with somatic illnesses in online support groups: Do the feared disadvantages actually occur?. Comput. Human Behav..

