# Supplementary material for: Automated gathering of real-world data from online patient forums can complement pharmacovigilance for rare cancers
Source: Sci Rep. 2022 Jun 20;12:10317. doi: 10.1038/s41598-022-13894-8 (PMC9209513; doi:10.1038/s41598-022-13894-8)
Supplement: Supplementary file 5 — Supplementary Information 5. [file 41598_2022_13894_MOESM5_ESM.docx]

Automated gathering of real-world data from online patient forums can complement pharmacovigilance for rare cancers

Anne Dirkson, dr. Suzan Verberne, Prof dr. Wessel Kraaij, Prof dr. Gerard van Oortmerssen and prof dr. Hans Gelderblom

# Appendix A Technical details of Methods

In Appendix A, we will elaborate on the technical details of how we preprocessed our data (section A.1), how we trained and evaluated models for extracting adverse drug events (ADE) (section A.2), how we trained and evaluated machine learning models to map the ADEs to the medical ontology SNOMED-CT (section A.3) and how we linked the reported ADEs to the medication the patient is reporting them for (section A.4). The Python code is publicly available at <https://github.com/AnneDirkson/CHyMer>.

## Appendix A.1. Data preprocessing

We preprocess the data with the pipeline [1] that includes replacing URLs and email addresses with the strings -URL- and -EMAIL- with regular expressions, lower-casing and tokenizing the text using NLTK, converting British to American English, expanding abbreviations to their full form (e.g., lol to laughing out loud) and expanding contractions (e.g., I’m to I am). Spelling mistakes were corrected using a combination of relative Levenshtein edit distance (i.e., how many insertions, deletions and replacements are necessary to change one word into another word relative to the length of the word) and cosine similarity based on a static (or context independent) word2vec language model [1]. A word2vec language model represents words based on how they are used, meaning that words used in similar contexts are closer together in the model and therefore have a lower cosine similarity. We exclude drug names in the FDA database of drugs (Downloaded from: <https://www.fda.gov/drugs/drug-approvals-and-databases/drugsfda-data-files>) from spelling correction to prevent common drug names replacing uncommon, similar drug names. Removing empty messages (567) and messages in a language other than English (1,493) left 121,516 messages. We also normalized drug names to their generic forms using the FDA database. We added the experimental names prior to approval by the FDA for novel GIST drugs manually (BLU-285 for Avapritinib and DCC-2618 for Ripretinib).

## Appendix A.2. Extracting ADEs from text

The task of extracting the words from a text that contain a certain concept (like an Adverse Drug Event) is called Named Entity Recognition. For Named Entity Recognition, entities are represented using the BIO scheme (B-Beginning, I-Inside and O-Outside). By default, this representation is not able to represent entities that overlap with other entities (e.g., *hand* and *foot pain*) or are split (*eyes* are feeling *dry*). These entities are coined discontinuous entities. We converted annotated data labels to the FuzzyBIO annotation scheme [2] to deal with these entity types. Discontinuous entities are transformed into continuous sequences that the BIO scheme can handle by annotating the non-entity words in between.

We make use of a state-of-the-art machine learning model for named entity recognition (BERT [3]) that has been trained on a large data set of English medical social media (EnDR BERT [4]). BERT models are a type of transfer learning model. Transfer learning models re-use a model trained on one (usually larger) data set as a starting point for training a model on another (usually smaller) data set to perform a task such as named entity recognition. For our model, we experimented with BERT models trained on biomedical text (i.e., PubmedBERT [5], BioBERT [6], and SciBERT[7]), but they did not do as well as EnDR BERT.

The initialization of such models is stochastic (i.e., has a degree of randomness). This can result in sub-optimal models [8]. To reduce this effect and create a more robust model, we use an ensemble of 10 models trained with different initialization seeds (1, 2, 4, 8, 16, 32, 64, 128, 256 and 512) following Weissenbacher *et al.* [8] and Miftahutdinov *et al.* [9]. We split our labelled data into training (80%), a validation (penultimate most recent 10% of the data) and a test set (most recent 10% of the data). We added a second publicly available data set of patient forum texts labelled for ADEs (CADEC [10]) to the training set. We also tried using PsyTAR [11] in order to increase the amount of data, but this was not beneficial. For each of the 10 models, we train for 3 or 4 epochs based on the results of the model on the first validation data. We use a one-cycle learning rate (LR) policy (max LR of 5^-5^) to train the models. We average the output of the 10 models using majority voting.

Table 2 reports the performance of the extraction of ADEs from text. The metrics used to calculate performance are recall, precision and the F_1_ score. Recall is the percentage of true positives that have been found. The precision is the percentage of true positives among the retrieved instances. The F_1_ score is a measure of the overall performance: it is the harmonic mean of precision and recall.

Here, tokens are relevant words that are part of an ADE. Our algorithm could retrieve 55.3% of all true positive tokens (‘Recall’) in a held-out test set and 72.3% of the retrieved tokens are true positives (‘Precision’). An entity is another term for the full concept e.g., ‘pain in chest’ is an entity while ‘pain’, ‘in’ and ‘chest’ are the tokens belonging to the entity. Our algorithm could retrieve 52.3% of all entities fully and 16.6% partially. On average, 69.5% of all retrieved entities were true positives. With this performance, our model performs better than state-of-the-art models on this task [12], [13]. It does still fall below human performance (average mutual F_1_= 0.80).

Besides a good performance, a model needs to be able to find entities that it has not seen previously in the training data [14]. We find that 40.2% of the true positive entities that our model finds are not present in the training data, indicating that our model is able to find novel entities in unlabeled data.

## Appendix A.3. ADE normalization

Normalization of adverse drug responses is the mapping of the text containing the ADE to concepts in an ontology (e.g., `cannot sleep' to Insomnia in SNOMED-CT). We use the current state-of-the-art method BioSyn [15] for normalizing the entities. We used the default parameters of BioSyn. BioSyn leverages BioBERT [6] (a BERT model trained on biomedical text) to rank all possible concept labels for an extracted ADE. The highest ranking label is selected. As was done in Sung *et al.* [15], we split composite mentions to separate mentions using heuristic rules by D’Souza and Ng [16] (e.g., non-familial breast and ovarian cancers into non-familial breast cancer and ovarian cancers). Our data does not contain annotations for normalization (i.e., the relevant concepts IDs for each ADE mention). We rely on three publicly available data sets for training our normalization model: CADEC [10], PsyTAR [11] and the Clinical Findings subset of the COMETA corpus [17].

We choose a curated subset of SNOMED, the CORE Problem List Subset as our target ontology. We try to map the concepts in the data sets to synonymous concepts in the CORE subset if possible. We do so by checking for a direct mapping in the community-based mappings in BioPortal [18] between the original concept. We also map the concept to its parent if the parent is in the CORE (e.g., ‘moderate anxiety’ to ‘anxiety’). As target concepts, we include all concepts of the CORE subset. SNOMED concepts present in the training data that could not be mapped to a CORE concept and SNOMED concepts present in the registration trial data that could not be mapped to a CORE concept as candidates. We also removed all concepts that are not in the Clinical Findings of SNOMED CT (e.g., Procedures like knee replacement). This results in a total of 5813 concepts. To employ the BioSyn method, we need to collect all synonyms of target SNOMED concepts. Synonyms for each concept are retrieved from the community based mappings in BioPortal [18] using the REST API and from the UMLS using pymedtermino [19].

The performance of the normalization model is shown in Table 3. On average, the model could accurately label 64.5% of the ADEs when tested on a different data set than those on which the model was trained. For an additional 14.6% of the cases, the correct label was included in the top 5. We manually inspected these cases and found that 53 of 100 randomly selected cases, we would consider the first label to be correct or even better than the gold label. For example, the mention ‘severe abdominal pain’ has the gold label ‘painful’ (i.e., the label given by humans) and the predicted label ‘abdominal pain’. Moreover, we inspected 100 random cases in which the correct label was not included in the top 5 and found that for 36 of those we would consider the predicted label as correct. Thus, the performance in Table 3 is likely an underestimation.

One concern is the propagation of errors in the pipeline (i.e., errors from extraction will influence normalization). Previous work has shown that ADE normalization is primarily hampered by errors made during extraction [13]. To assess the pipeline end-to-end, we manually inspect 100 of the found ADEs in the GIST data. As can been seen in Table 4, we find that 67 of the 100 cases is correct, where 22 of the 100 are incorrect due to extraction errors prior to normalization. Thus, extraction appears to still be the major bottleneck.

Another concern is that the normalization model should be able to predict new types of ADE that are absent in the training data. The BioSyn model should theoretically be able to do so because all the concepts of the SNOMED CT are considered as possible targets for mapping. Our normalization model is indeed able to predict classes that were not part of the training data at an Accuracy@1 of 0.417 on average and an Accuracy@5 of 0.612 on average for the external data sets. On our own GIST data, we also see empirically that 15.0% of the predicted concepts are not part of the training data.

Manual analysis of the predicted concepts in the GIST data revealed that some ADEs for tyrosine kinase inhibitors (TKIs) (e.g. split nails, hair color change, and hand-foot syndrome) were not included in the target concepts. We added 5 concepts and 2 synonyms to existing concepts manually to improve normalization.

Appendix A.4. Linking ADEs to medication

First, we identify all drugs mentioned in each message using a dictionary based on the RxNORM [20]. During preprocessing, we already converted all brand names to their generic equivalents (e.g., Gleevec to Imatinib).

We use heuristic rules to determine which drug is linked to each ADE. Whenever possible, we select the drug mentioned prior to the ADE in the message. If there is none, we select the drug mentioned after the ADE in the message. If there are no drugs mentioned in the message, we select the first drug mentioned in the conversational thread prior to the message. These rules were determined based on further manual annotation of our annotated subset by the first author. In some cases, it was not clear which drug the patient believed was causing the ADE and these cases were excluded. In this data set, our rules were 93% accurate if we restricted the possible drugs for linking to a predetermined list (Imatinib, Sunitinib, Regorafenib, Avapritinib, Ripretinib, Nilotinib, Pazopanib, Ponatinib, Sorafenib).

**Table 2** The performance of the ADE extraction model on the held-out test set. Here the entity-level performance is lenient: an entity is regarded as true positive if at least one token has been retrieved correctly.

|  | F_1_ | Precision | Specificity | Sensitivity/Recall |
| --- | --- | --- | --- | --- |
| Token level performance | 0.626 | 0.723 | 0.995 | 0.553 |
| Entity level performance | 0.716 | 0.739 | 0.998 | 0.695 |

**Table 3** The performance of our normalization model on a held-out data set. As the normalization model provides a ranking of candidate labels, Acc @1 and Acc @5 indicates the percentage of cases with the correct label in the top 1 and top 5 respectively. The bold numbers indicate mean values

| Trained on | Tested on | Acc @1 | Acc @5 |
| --- | --- | --- | --- |
| CADEC & COMETA | PsyTAR | 0.586 | 0.771 |
| COMETA & PsyTAR | CADEC | 0.663 | 0.807 |
| CADEC & PsyTAR | COMETA | 0.688 | 0.795 |
|  |  | **0.645** | **0.791** |

**Table 4** Manual analysis of 100 randomly selected found ADEs in the GIST data

| Category | Frequency | Example |
| --- | --- | --- |
| Correct concept | 67 | - |
| Extraction errors | 22 | ‘feet’, ‘nose’, ‘losing’ |
| Predicted concept is related | 6 | ‘kidney issues’ instead of ‘nephrosis’ |
| No SNOMED equivalent | 2 | ‘comfy eyes, woozy face’ |
| Wrong but no clear reason | 2 |  |

## References

[1] A. Dirkson, S. Verberne, A. Sarker, and W. Kraaij, “Data-Driven Lexical Normalization for Medical Social Media,” *Multimodal Technol. Interact.*, vol. 3, no. 3, p. 60, Aug. 2019.

[2] A. Dirkson, S. Verberne, and W. Kraaij, “FuzzyBIO: A Proposal for Fuzzy Representation of Discontinuous Entities,” in *Proceedings of the 12th International Workshop on Health Text Mining and Information Analysis*, 2021, pp. 77–82.

[3] J. Devlin, M.-W. Chang, K. Lee, and K. Toutanova, “BERT: Pre-training of Deep Bidirectional Transformers for Language Understanding,” in *Proceedings of the 2019 Conference of the North {A}merican Chapter of the Association for Computational Linguistics: Human Language Technologies, Volume 1 (Long and Short Papers)*, 2019.

[4] E. Tutubalina, I. Alimova, Z. Miftahutdinov, A. Sakhovskiy, V. Malykh, and S. Nikolenko, “The Russian Drug Reaction Corpus and Neural Models for Drug Reactions and Effectiveness Detection in User Reviews,” *Bioinformatics*, 2020.

[5] Y. Gu *et al.*, “Domain-specific language model pretraining for biomedical natural language processing,” *arXiv*, pp. 1–24, 2020.

[6] J. Lee *et al.*, “BioBERT: a pre-trained biomedical language representation model for biomedical text mining,” *Bioinformatics*, vol. 1, no. 36, pp. 1-, Jan. 2019.

[7] I. Beltagy, A. Cohan, and K. Lo, “SciBERT: Pretrained Contextualized Embeddings for Scientific Text,” in *Proceedings of the 2019 Conference on Empirical Methods in Natural Language Processing and the 9th International Joint Conference on Natural lanugage processing (EMNLP-IJCNLP)*, 2019, pp. 3615–3620.

[8] D. Weissenbacher, A. Sarker, A. Klein, K. O’connor, A. Magge, and G. Gonzalez-Hernandez, “Deep neural networks ensemble for detecting medication mentions in tweets,” *J. Am. Med. Informatics Assoc.*, vol. 26, no. 12, pp. 1618–1626, Nov. 2019.

[9] Z. Miftahutdinov and E. Tutubalina, “Deep Neural Models for Medical Concept Normalization in User-Generated Texts Deep Neural Models for Medical Concept Normalization in User-Generated Texts,” in *Student Research Workshop ACL 2019*, 2019.

[10] S. Karimi, A. Metke-Jimenez, M. Kemp, and C. Wang, “Cadec: A corpus of adverse drug event annotations,” *J. Biomed. Inform.*, vol. 55, pp. 73–81, Jun. 2015.

[11] M. Zolnoori *et al.*, “The PsyTAR dataset: From patients generated narratives to a corpus of adverse drug events and effectiveness of psychiatric medications.,” *Data Br.*, vol. 24, Jun. 2019.

[12] A. Magge *et al.*, “DeepADEMiner: A Deep Learning Pharmacovigilance Pipeline for Extraction and Normalization of Adverse Drug Effect Mentions on Twitter,” *JAMIA*, p. 2020.12.15.20248229, Dec. 2020.

[13] D. Weissenbacher *et al.*, “Overview of the Fourth Social Media Mining for Health (#SMM4H) Shared Task at ACL 2019,” in *Proceedings ofthe 4th Social Media Mining for Health Applications (#SMM4H) Workshop & Shared Task*, 2019, pp. 21–30.

[14] E. Tutubalina, A. Kadurin, and Z. Miftahutdinov, “Fair Evaluation in Concept Normalization : a Large-scale Comparative Analysis for BERT-based Models,” in *COLING 2020*, 2020.

[15] M. Sung, H. Jeon, J. Lee, and J. Kang, “Biomedical Entity Representations with Synonym Marginalization,” in *Proceedings of the 58th Annual Meeting of the Association for Computational Linguistics*, 2020, pp. 3641–3650.

[16] J. D. ’ Souza and V. Ng, “Sieve-Based Entity Linking for the Biomedical Domain,” in *53rd Annual Meeting ofthe Association for Computational Linguistics and the 7th International Joint Conference on Natural Language Processing*, 2015, pp. 297–302.

[17] M. Basaldella, F. Liu, E. Shareghi, and N. Collier, “COMETA: A Corpus for Medical Entity Linking in the Social Media,” in *Proceedings of the 2020 Conference on Empirical Methods in Natural Language Processing*, 2020, pp. 3122–3137.

[18] N. F. Noy, N. Griffith, and M. A. Musen, “Collecting community-based mappings in an ontology repository,” in *Lecture Notes in Computer Science (including subseries Lecture Notes in Artificial Intelligence and Lecture Notes in Bioinformatics)*, 2008, vol. 5318 LNCS, pp. 371–386.

[19] J. B. Lamy, A. Venot, and C. Duclos, “PyMedTermino: An open-source generic API for advanced terminology services,” *Stud. Health Technol. Inform.*, vol. 210, pp. 924–928, 2015.

[20] U.S. National Library of Medicine, “RxNorm.” [Online]. Available: https://www.nlm.nih.gov/research/umls/rxnorm/.
